# Effect of *Microsporidia MB* infection on the development and fitness of *Anopheles arabiensis* under different diet regimes

**DOI:** 10.1186/s13071-024-06365-8

**Published:** 2024-07-09

**Authors:** Godfred Yaw Boanyah, Lizette L. Koekemoer, Jeremy K. Herren, Tullu Bukhari

**Affiliations:** 1 International Centre of Insect Physiology and Ecology, Nairobi, Kenya; 2 Wits Research Institute for Malaria, Faculty of Health Sciences, University of the Witwatersrand, Johannesburg, South Africa; 3 Centre for Emerging Zoonotic & Parasitic Diseases, Division of the National Health Laboratory Service, National Institute for Communicable Diseases, Johannesburg, South Africa

**Keywords:** *Microsporidia MB*, Larval diet, Adult diet, Life history traits

## Abstract

**Background:**

*Microsporidia MB* (*MB*) is a naturally occurring symbiont of *Anopheles* and has recently been identified as having a potential to inhibit the transmission of *Plasmodium* in mosquitoes. *MB* intensity is high in mosquito gonads, with no fitness consequences for the mosquito, and is linked to horizontal (sexual) and vertical (transovarial) transmission from one mosquito to another. Maximising *MB* intensity and transmission is important for maintaining heavily infected mosquito colonies for experiments and ultimately for mosquito releases. We have investigated how diet affects the *MB-Anopheles arabiensis* symbiosis phenotypes, such as larval development and mortality, adult size and survival, as well as *MB* intensity in both larvae and adults.

**Methods:**

F_1_ larvae of G_0_ females confirmed to be *An. arabiensis* and infected with *MB* were either combined (group lines [GLs]) or reared separately (isofemale lines [IMLs]) depending on the specific experiment. Four diet regimes (all mg/larva/day) were tested on F_1_ GLs: Tetramin 0.07, Tetramin 0.3, Gocat 0.3 and Cerelac 0.3. GLs reared on Tetramin 0.3 mg/larva/day were then fed either a 1% or 6% glucose diet to determine adult survival. Larvae of IMLs were fed Tetramin 0.07 mg and Tetramin 0.3 mg for larval experiments. The mosquitoes in the adult experiments with IMLs were reared on 1% or 6% glucose.

**Results:**

Amongst the four larval diet regimes tested on *An.* *arabiensis* development in the presence of *MB*, the fastest larval development highest adult emergence, largest body size of mosquitoes, highest prevalence and highest density of *MB* occurred in those fed Tetramin 0.3 mg/larva/day. Although adult *MB-*positive mosquitoes fed on 6% glucose survived longer than *MB*-negative mosquitoes, there was no such effect for those fed on the 1% glucose diet. Development time, wing length and adult survival were not significantly different between *MB-*infected and uninfected *An. arabiensis* fed on the Tetramin 0.07 mg/larva/day diet, demonstrating that the *MB*-conferred fitness advantage was diet-dependent.

**Conclusions:**

*Microsporidia MB* does not adversely impact the development and fitness of *An. arabiensis*, even under limited dietary conditions. The diet regime of Tetramin 0.3 mg/larva/day + 6% glucose for adults is the superior diet for the mass rearing of *MB*-infected *An. arabiensis* mosquitoes. These results are important for rearing *MB-*infected *An. arabiensis* in the laboratory for experiments and the mass rearing required for field releases.

**Graphical Abstract:**

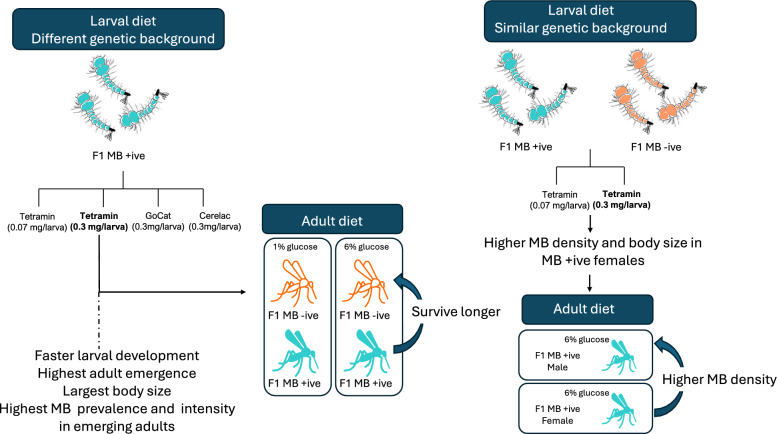

**Supplementary Information:**

The online version contains supplementary material available at 10.1186/s13071-024-06365-8.

## Background

Despite the massive effort and investment directed towards malaria control through the improvement of healthcare systems, vector control and development of drugs for treatment of the disease, malaria remains an important public health problem in the world, especially in Africa [1, 2]. In 2023, there were 608,000 deaths due to malaria worldwide, with children being the most affected group [3]. Malaria deaths increased by 12% in 2020 compared to 2019, likely due to the challenges imposed on the operation of malaria control programmes by the COVID-19 pandemic [4]. Malaria transmission in the past three decades has drastically declined due to the adoption of long-lasting insecticide-treated bed nets (LLINs) and indoor residual spraying (IRS) [2]. However, the combined impact of climate change on mosquito vectors [5], insecticide resistance [6, 7] behavioural changes, such as early biting, and other factors have started to reverse the gains that had been made and are a threat to case burden reduction targets [1, 8, 9]. Considering these developments, there is an urgent need for alternative vector control approaches.

Symbiosis is a term that defines a close physical or ecological interaction between two different species. This association could be beneficial to one (commensal) or both (mutual) of the organisms involved, detrimental to one of the organisms or at times have a neutral effect on the organisms. Endosymbionts are symbiotic organisms that reside within a host organism, either inside the host’s cells (intracellular) or outside the cells (extracellular) in multicellular hosts. Endosymbionts play a very crucial role in insect biology by enhancing both nutritional provision [10] and defense [11], affecting fitness either positively or negatively (e.g. [12], [13]). However, environmental factors such as temperature and nutrition determine the stability and intensity of these symbionts in their host insects and their ability to provide protective effects or cause harmful effects on the insects (e.g. [13]–16])

The nutritional adaptations and requirements of the aquatic larvae stage of *Anopheles* mosquitoes differ significantly from those of terrestrial adult-stage mosquitoes [16, 17]. The mouth brushes of the omnivorous scavenger larvae are used to collect suspended food particles from submerged surfaces [18]. Adult mosquitoes, on the other hand, gain energy mainly from plant sources, such as the nectar of flowers [19]. Several studies have investigated the effect of diet on *Anopheles* mosquitoes [19–21]. One notable finding of such studies is that the content of larval diet has a significant effect on both larval development and adult body size [22]. Moreover, restricting the food of mosquito larvae can result in negative effects on survival and development [23]. A study on *Anopheles arabiensis* found that fatty acid profiles in mosquitos were modified by the larval diet, which controlled mosquito size, phosphorus nutrition and population size [24].

Studies have shown that the reproductive capacity of mosquitoes is also affected by the type and quantity of diet they feed on [20, 25]. Variations in the concentrations of sugar derived from plants have been demonstrated to impact the lifespan and reproductive capabilities of adult female *Anopheles gambiae*. Longevity, body size and biting frequency are determined by the quality and quantity of food consumed by the mosquito in its lifetime [21]. This, in turn, affects the vectorial capacity or disease transmission potency of the mosquito [21, 26]. Adult *Anopheles stephensi* mosquitoes fed a lower abundance of larval diet and infected with *Plasmodium falciparum* exhibited a delay in parasite development and an increase in adult mortality, resulting in the mosquito requiring a longer period to become infectious [27]. The prevalence and intensity of adult infection in *Anopheles coluzzii* were significantly impacted by the larval diet they received during their larval stage when infected with *Plasmodium berghei* [28].

Nutrition is well recognised as a crucial factor in influencing the interactions between a host and its symbiotic organisms [29, 30]. Diet composition significantly influences the interaction between *Wolbachia*, which are maternally inherited bacterial endosymbionts in insects, and the host's diet selection [29, 31, 32]. This, in turn, affects the intensity of *Wolbachia* and its impact on the host's longevity and fertility [33]. There are three processes through which the host regulates endosymbionts [33]. The first mechanism suggests that the intensity of the symbionts is frequently controlled by the dietary requirements of the host; for example, hosts may have a varying intensity of symbionts depending on the quality of their food [34].

The endosymbiotic microsporidium *Microsporidia MB* has been found to prevent *An. arabiensis* in Kenya from transmitting the *Plasmodium* parasite [35]. *Microsporidia* are obligate intracellular eukaryotes that are related to fungi [36]. *Microsporidia MB* are localised in the gonads of *An. arabiensis* and are transmitted between mosquitoes by two routes: horizontal transmission, which occurs through mating, and vertical transmission from mother to offspring [35, 37]. The intensity of *Microsporidia MB* infection is important for vertical transmission, and possibly for horizontal transmission [35]. However, how environmental factors such as diet affect *Microsporidia MB* transmission and aspects of the host-symbiont interaction are unknown.

A high intensity of *Microsporidia MB* infection in *Anopheles* mosquitoes is linked to higher vertical transmission [35]. Hence, to advance the development of *Microsporidia MB* as a malaria control tool, it is important to understand the factors that contribute to the maintenance of a high prevalence and intensity of *Microsporidia MB* in mosquito colonies. In addition, the spread of *Microsporidia MB* will be affected by impacts on host fitness under a variety of natural conditions. We have investigated the effects of different diet regimes on *Microsporidia MB* infection parameters in *An. arabiensis* and determined how different diet regimes affect *An. arabiensis* host fitness in the presence of *Microsporidia MB*.

## Methods

### Mosquitoes, species identification and *Microsporidia MB* screening

The progeny of field-collected mosquitoes was used for this study. Blood-fed and indoor resting *Anopheles* mosquitoes were aspirated from houses around the Ahero irrigation scheme, Kisumu County, Kenya. The mosquitoes were transferred to the laboratory (International Centre Of Insect Physiology And Ecology [Icipe], Thomas Odhiambo Campus, Mbita, Kenya) in cages (30 × 30 × 30 cm) covered with a moist towel with mosquitoes provided access to 6% glucose solution. In the laboratory, oviposition was induced by transferring an individual female mosquito (G_0_) to a 1.5-ml Eppendorf tube that had been lined with filter paper and filled with 100 µl of water [38]. The eggs were dispensed into the water in larval trays/tubs (21 × 15 × 8.5 cm) kept in semi-field conditions in screened houses. The G_0_ females were screened by PCR to determine the species and presence of *Microsporidia MB* following established protocols, briefly described in the following text. The F_1_ larvae of G_0_ females were confirmed to be *An. arabiensis* and infected with *Microsporidia MB* were either combined (group lines [GLs]) or reared separately (isofemale lines [IMLs]) depending on the experiments. Similarly, the larvae of females confirmed to be *An. arabiensis* but not infected with *Microsporidia MB* were combined or reared separately to serve as controls for the experiments. All experiments were carried out on either F_1_ larvae or adults.

After oviposition, wild-caught females were screened using morphological characteristics [39] and PCR methods [40]. Quantitative PCR (qPCR) was performed by first isolating DNA using the ammonium acetate protein precipitation technique [35]. The DNA samples thus obtained were analysed to determine whether they were infected with *Microsporidia MB* using the specific primers MB18SF (CGCCGG CCGTGAAAAATTTA) and MB18SR (CCTTGGACGTG GGAGCTATC) that target the *Microsporidia MB* 18S ribosomal RNA (rRNA) region [35]. The PCR analyses were carried out in a reaction volume of 10 µl containing 2 µl of HOT FIREPol Blend Master Mix Ready-To-Load (Solis Biodyne, Tartu, Estonia), 0.5 µl of forward and reverse primers at a concentration of 5 pmol/µl, 2 µl of the DNA template and 5 µl of nuclease-free water. The thermocycling protocol consisted of an initial denaturation step at 95 °C for 15 min, followed by 35 cycles of denaturation at 95 °C for 1 min, annealing at 62 °C for 90 s and extension at 72 °C for 60 s, with a final extension at 72 °C for 5 min. *Microsporidia MB-*positive samples underwent relative qPCR analysis to measure infection levels [35]. The qPCR utilised the MB18SF/MB18SR primers, with normalisation performed using the *Anopheles* ribosomal S7 gene (primers: S7F [TCCTGGAGCTGGAGATGAAC] and S7R [GACGGGTCTGTACCTTCTGG]) as the reference gene. The PCR analyses were carried out in a reaction volume of 10 µl containing 2 µl of HOT FIREPol EvaGreen HRM no ROX Mix Solis qPCR Master Mix (Solis Biodyne), 0.5 µl of forward and reverse primers at a concentration of 5 pmol/µl, 2 µl of DNA template from *Microsporidia MB*-positive samples and 5 µl of nuclease-free PCR. The thermocycling protocol consisted of an initial denaturation step at 95 °C for 15 min, followed by 35 cycles of denaturation at 95 °C for 1 min, annealing at 62 °C for 90 s and extension at 72 °C for 60 s. Ultimately, the melting curves were produced by a melting step, utilising temperatures ranging from 65 °C to 95 °C. The PCR and qPCR were performed using a MIC qPCR cycler (Bio Molecular Systems, Upper Coomera, Australia). Confirmation was obtained for each sample, indicating the presence of the distinctive melt curve that is related to the *Microsporidia MB* MB18SF/MB18SR primers.

### Effect of *Microsporidia MB* on developmental fitness under different larval diet regimes

The selection of diet regimes was based on previous literature [20, 41, 42]. In total, there were eight treatments/diet regimes (Additional file 1, Figure 2), and each treatment was replicated 3 times. Each replicate consisted of 60 unfed 24 h-old *An. arabiensis* larvae placed in a larval tray (21 × 15 × 8.5 cm) filled with 1 l of distilled water. The larvae came from either *Microsporidia MB*-infected or uninfected GL lines. Three types of diets were tested [41]: (i) TetraMin® baby fish diet (TD; Tetra GmbH, Melle, Germany), Cerelac® baby diet (CD) and diary powdered milk (Nestle S.A., Vevey, Switzerland) and Gocat® diet (GD; Purina®, St. Louis, MO, USA). The nutritional content of each diet is given in Additional file 2, Table 1. Two doses of TD were tested, namely 0.3 mg/larva/day and 0.07 mg/larva/day, and one dose of CD and GD was tested, namely 0.3 mg/larva/day. The size of the flakes or granules was reduced by grinding, a routine larval-rearing practice in the laboratory [28]. The TD 0.3 mg/larva/day diet was used as the reference diet and served as a positive control diet regime, while the TD 0.07 mg/larva/day diet was tested to determine how low diet availability during larval development influences the effect of *Microsporidia MB* on mosquito fitness [43]. The number of dead larvae was recorded and removed daily. The number of pupae was also recorded daily, and these were transferred to emergence cages. The diet added to the larval trays was adjusted to the number of larvae that remained in the larval trays. The emerging adults from each treatment group were fed on 6% glucose solution ad libitum. Twenty adults (10 males and 10 females) were harvested on day 3 from each group and used to determine wing length as a proxy for body size [44, 45]; the remaining mosquitoes were screened for *Microsporidia MB* presence and intensity. The wing length was measured in millimetres using a Dino-Lite® Premier handheld microscope at a magnification of 32.1× (Huatang Optical Industry Co., Ltd, Taiwan). The regime that resulted in the highest survival and *Microsporidia MB* intensity was used for all the other experiments unless otherwise stated.

### Effect of *Microsporidia MB* on adult mosquito survival under different adult diet regimes

Larvae of *Microsporidia MB*-infected and -uninfected GLs were reared on Tetramin 0.3 mg/larva/day; the diet regime was selected based on results from previous experiments (Additional file Figure 3). At least 80 adults from either *Microsporidia MB*-infected or -uninfected GLs were used in the experiment. The adult mosquitoes (< 1 day old) were divided into two treatment groups (*n* = 40) and placed in separate cages (15 × 15 × 15 cm). In one cage, mosquitoes had ad libitum access to 1% glucose solution and in the second cage mosquitoes had ad libitum access to 6% glucose solution (Additional file Figure 3). The glucose solution was provided in a vial and mosquitoes could feed through a paper towel wick. The vial, glucose solution and wick were replaced every 2 days. The above procedure was conducted concurrently for both *Microsporidia MB*-positive and -negative GLs. Daily adult mortality was recorded. The dead mosquitoes were screened for *Microsporidia MB*. The prevalence and densities of *Microsporidia MB* infection in mosquitoes from each diet regime were recorded. This experiment was conducted in two rounds, with three replicates for each diet treatment group in each round.

### Effect of larval and adult diet quantity on the intensity of *Microsporidia MB* infection in *An. arabiensis* adult with a similar genetic background.

To determine if the effect of different quantities of larval and adult diet on *Microsporidia MB* prevalence and intensity is influenced by the genetic background of the mosquitoes, experiments were conducted with IMLs (Additional file Figure 4). This study was implemented to enhance detection of variations in intensity among mothers as data suggest that the intensity of endosymbionts can be inherited. For this experiment (Additional file Figure 4A), the two larval diet regimes from TD, 0.07 mg and 0.3 mg per larva/day, were referred to as the low and high nutritional diet, respectively [43]. Larvae from *Microsporidia MB-*positive IMLs were divided into two trays and reared on either the low or high nutritional diet.

Dead larvae were removed daily and recorded. Pupae were also collected daily and placed in a 15 × 15 × 15-cm cage. The adults that emerged were fed on 6% glucose solution ad libitum and harvested to quantify *Microsporidia MB* intensity when 3 days old. This experiment was carried out in nine replicates, with each replicate consisting of at least 20 unfed 24-h-old *An. arabiensis* larvae from the same mother placed in a larval tray (21 × 15 × 8.5 cm) with 1 l of distilled water.

To determine if the effect of adult diet on *Microsporidia MB* intensity was influenced by genetic background, larvae from IMLs were reared on 0.3 mg TD/larva. The IMLs that produced at least 40 adults were used for this experiment (Additional file Figure 4B). The adults were split in two groups and placed in separate cages (15 × 15 × 15 cm). Mosquitoes in one cage were fed on 1% glucose solution and those in the second cage were fed on 6% glucose solution ad libitum. The sugar solution was replaced with a fresh one every 2 days. On day 14, the adult mosquitoes were harvested to quantify *Microsporidia MB* using qPCR. A period of 14 days was chosen to ensure the accurate measurement of the impact of the adult diet.

### Data analysis

Kaplan–Meier survival analysis and Cox regression were used to determine the effect of different diet regimes on larval development and adult survival. Prevalence and adult emergence data was arcsine transformed and analysed using the Tukey’s honestly significant difference (HSD) test of analysis of variance (ANOVA) to determine the best-fit diet regime. Non-parametric Mann–Whitney U and Kruskal–Wallis tests were used to compare the *Microsporidia MB* intensities of the adult and larval diet treatment groups. Phenotypic characteristics, such as length of wing, were tested for significant variation within and across treatment groups using the generalised linear model (GLM) following gamma distribution. R software version 4.1.2 (R Foundation for Statistical Computing, Vienna, Austria) was used for analysis with a *P*-value < 0.05 considered to be significant at a 95% confidence interval.

## Results

### Prevalence and Intensity of *Microsporidia MB* under different larval diet regimes

**Fig. 1:**
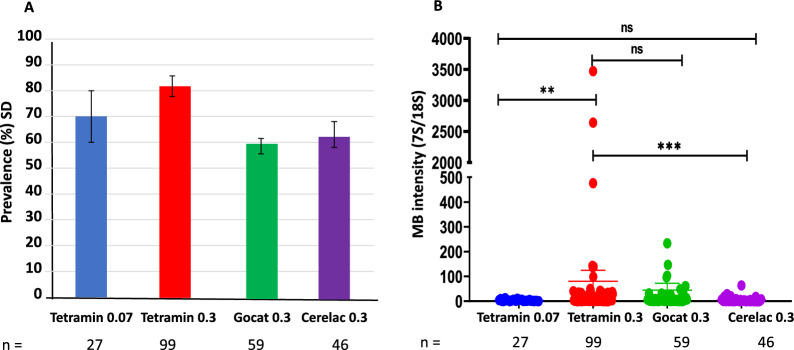
*Microsporidia MB* prevalence and intensity under different larval diet regimes. **a** Prevalence (%) of *Microsporidia MB* in 3-day-old F_1_ adults reared on different diet regimes. Error bars represent the standard deviation. **b** Relative *Microsporidia MB* intensity in 3-day-old F_1_ adults reared on different diet regimes using qPCR. Bar represents a significant difference between the diet regimes. There were 3 independent biological replicates, with each replicate comprising 60 larvae. The experimental design is shown in Additional file 2. Asterisks indicate the level of significance (*P < 0.05, **P < 0 .001, ***P < 0.0001); ns indicates no significance.* qPCR* Quantitative PCR

There was no significant difference in *Microsporidia MB* prevalence across the four diet regimes (ANOVA after arcsine transformation of the data, *F *= 2.655, df = 3, *P* = 0.185) (Fig. 1a). This result showed that larval diet regimes did not affect the prevalence of *Microsporidia MB* in emerging adults. The intensity of *Microsporidia MB* was affected by the different larval diet regimes (Kruskal–Wallis test, *X*^2^ = 22.85, df = 3, *P*-value < 0.0001). Multiple pairwise comparisons using Wilcoxon rank sum test with continuity correction showed that 3-day-old adults emerging from the larvae feeding on TetraMin (TD) 0.3 mg/larva/day had a higher intensity of *Microsporidia MB* than those feeding on TD 0.07 mg/larva/day (*P* = 0.007) and Cerelac (CD) 0.3 mg/larva/day (*P* < 0.001) (Fig. 1b). There was no significant difference between *Microsporidia MB* intensity between larvae fed TD 0.3 and Gocat (GD) 0.3 mg/larva/day diets. Furthermore, there was a significant difference in *Microsporidia MB* intensity between larvae fed on GD 0.3 mg/larva/day and those fed on CD 0.3 mg/larva/day (*P *< 0.006); however, there was no difference in *Microsporidia MB* intensity between larvae fed TD 0.07 mg/larva/day and those fed CD 0.3 mg/larva/day (*P* > 0.254).

### Effect of *Microsporidia MB* on larval mortality and pupation under different larval diet regimes

**Fig. 2:**
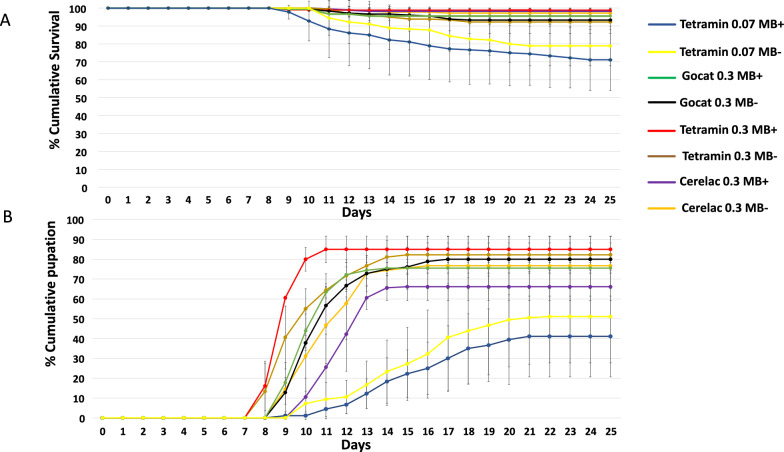
Effect of *Microsporidia MB* on mortality and larval development under different larval diet conditions. **a** Effect of *Microsporidia MB* on larval mortality under different diet regimes, **b** effect of *Microsporidia MB* on larval developmental time under different diet regimes. Error bars represent standard deviations

The Kaplan–Meier survival analysis showed that larval mortality was highest under the TD 0.07 mg/larva/day regime, being 26% in *Microsporidia MB*-infected and 28.33% in *Microsporidia MB*-uninfected larvae, out of the total 180 larvae in three biological replicates (*n* = 60 larvae) in each replicate. Mortality in *Microsporidia MB*-infected and -uninfected was 4.45% and 6.67%, respectively, under the GD 0.3 mg/larva/day diet and 1.70% and 2.78%, respectively, under the CD 0.3 mg/larva/day diet (Fig. 2a). There was a significantly higher survival under the TD 0.3 mg/larva/day diet, with a mortality of 1.67% in *Microsporidia MB*-infected larvae and 15% in *Microsporidia MB*-uninfected larvae (*X*^2 ^= 15, df = 1, *P* < 0.001). Under the TD 0.3 mg/larva/day diet regime and GD diet regime, *Microsporidia MB* significantly reduced larva mortality (*X*^2^ = 15, df = 1, *P* < 0.001 and *X*^2^ = 5.43, df = 1, *P* = 0.012, respectively).

As previously observed [35], we noted that *Microsporidia MB-*infected larvae developed faster than their uninfected counterparts. The enhancement of *Microsporidia MB* growth rate was diet dependent. *Microsporidia MB-*infected larvae developed significantly faster than their uninfected counterparts only when fed on the TD 0.3 and GD 0.3 mg/larva/day diet regimes (Hazard ratio (HR) = 1.7, 95% CI = 1.4–2.2, *P* < 0.001 and HR = 1.4, 95% CI = 1.1–1.7, *P* < 0.01, respectively). Under the TD 0.3 mg/larva/day diet regime, the median (± standard deviation) developmental time was shorter for *Microsporidia MB-*infected larvae than for their uninfected controls (9 ± 0.06 vs 10 ± 0.15 days, respectively) (Fig. 2b). The hazard ratio of 1.7 (95% CI, 1.4–2.2; *P*<0.001) showed that *Microsporidia MB*-infected larvae developed 1.7 days faster than the uninfected controls*.* Under the GD 0.3 mg/larva/day diet regime, the median development time for *Microsporidia MB-*infected larvae was also shorter than that of the uninfected larvae (10 ± 0.12 vs 11 ± 0.14 days, respectively). The median developmental time for *Microsporidia MB*-infected and -uninfected larvae fed CD 0.3 mg/larva/day was 12 ± 0.12 days and 11 ± 0.21 days, respectively. However, in larvae fed the TD 0.07 mg/larva/day diet there was little difference in the developmental period between the *Microsporidia MB*-infected larvae and the control larvae (15 ± 0.52 days and 15 ± 0.72 days, respectively).

**Fig. 3:**
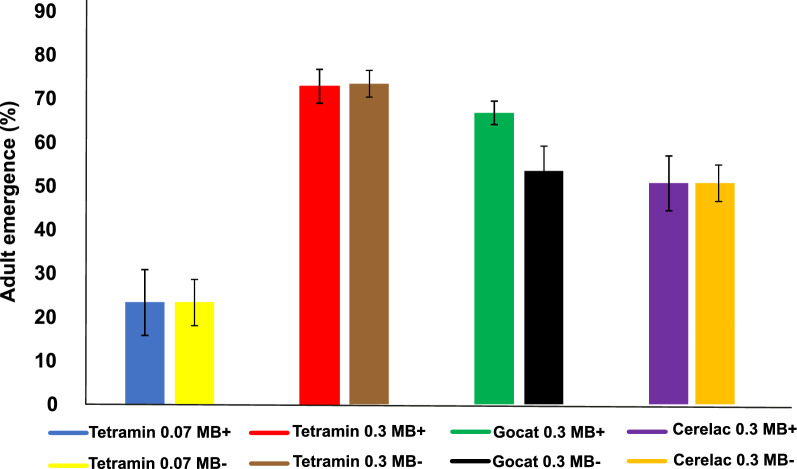
*Microsporidia MB's* on adult emergence under the different larval diet regimes. The data shown are the percentage of larvae that developed into adult mosquitoes from larvae fed the various diet regimes and represent the total number of adults that emerged from the collected pupae from each diet. Error bars represent standard deviation

The different diets affected the emergence of pupated larvae into adults (*F* = 4.66, df = 3, *P *= 0.005) (Fig. 3). It must be noted that not all pupae emerged into adult mosquitoes, and this was especially noted for those fed the TD 0.07 mg/larva/day diet; this accounted for the difference in pupae and emerged adult mosquito numbers. However, for each treatment, there was no significant difference between *Microsporidia MB*-infected GLs and control GLs (*F* = 4.66 , df = 3, *P* = 0.99).

### Effect of *Microsporidia MB* on adult mosquito survival under different adult diet regimes

**Fig. 4:**
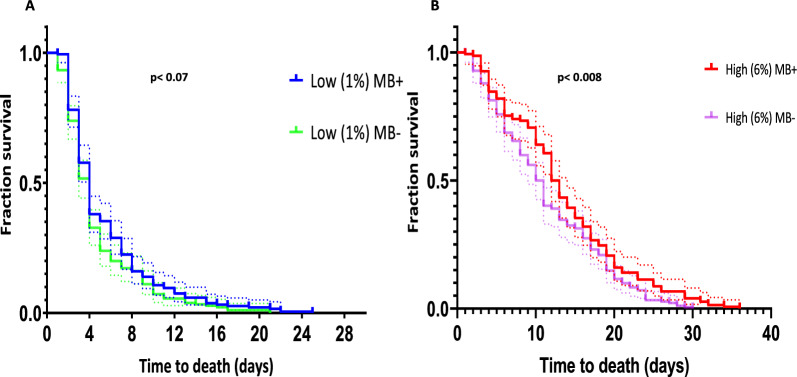
Effect of *Microsporidia MB* on adult mosquito survival time under different adult diet quantity regimes. **a** 1% glucose diet, **b** 6% glucose diet

The median survival time for *Microsporidia MB*-infected and uninfected adult mosquitoes from GLs reared on the same larval diet regime (TD 0.3 mg/larva/day), when fed a 1% glucose diet (Fig. 4a), was 4 ± 0.17 days and 4 ± 0.18 days, respectively. There was no significant difference in survival between *Microsporidia MB*-infected and -uninfected adults (*X*^2^ = 2.95, df = 1, *P* = 0.073).

On the other hand, among those adult mosquitoes fed on the 6% glucose diet (Fig. 4b), *Microsporidia MB-*infected mosquitoes survived significantly longer than their uninfected counterparts (*X*^2^ = 5.84, df = 1, *P * = 0.007). The median survival time for the *Microsporidia MB-*infected adults was 12 ± 0.49 days, compared to 10 ± 0.47 days for their uninfected counterparts.

### Effect of larval and adult diet quantity on *Microsporidia MB* intensity in IMLs

**Fig. 5:**
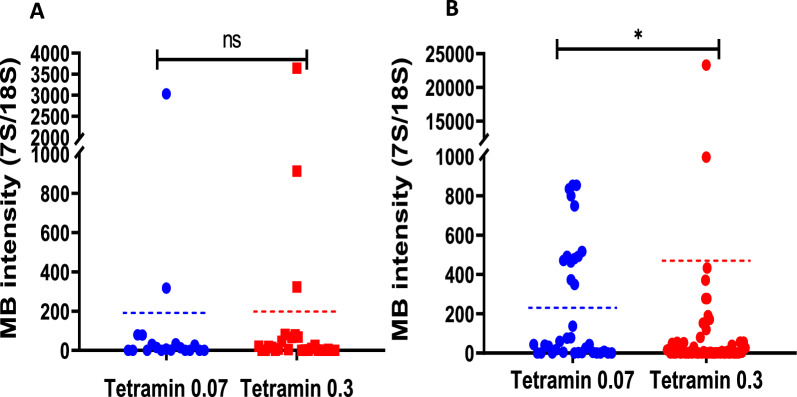
*Microsporidia MB* intensity under different larval diet quantity (**A**) *Microsporidia MB*intensity of males and (**B**) *Microsporidia MB*intensity of female adults that emerged from larvae reared on Tetramin 0.07 mg/larva/d and 0.3 mg/larva/d. The dotted lines represent the mean intensity of each diet regime

We compared *Microsporidia MB* intensity in adult mosquitoes emerged from IML larvae which had been fed on the TD 0.07 mg/larva/day and TD 0.3 mg/larva/day diets, respectively (Fig. 5b). The intensity of female mosquitoes was significantly affected by the two larval diets, with the latter mosquitoes having the higher *Microsporidia MB* intensity than those on the low nutritional diet ( *X*^2^ = 6.38, *df* = 1, *P*-value = 0.011). Meanwhile, the adult male mosquitoes (Fig. 5a) did not show any significant difference in intensity between both diets ( *X*^2^= 0.30, *df* = 1, *P*-value = 0.584).

IMLs which were fed on the TD 0.07 mg/larva/day diet were shown to result in significantly smaller sized adult mosquitoes when compared to those fed the TD 0.3 mg/larva/day regime (*t *= 4.23, df = 1, *P* < 0.0001). Males fed on both diets had smaller body sizes than their female counterparts (*t* = 5.93, df = 1, *P* < 0.0001). *Microsporidia MB*-positive IML female mosquitoes had a significantly longer wing length under both the TD 0.07 mg/larva/day diet (*X*^2^ = 4.18, *df* = 1, *P*= 0.040) and the TD 0.3 mg/larva/day diet ( *X*^2^= 23.21, *df* = 1, *P* < 0.0001) than their male counterparts.

**Fig. 6:**
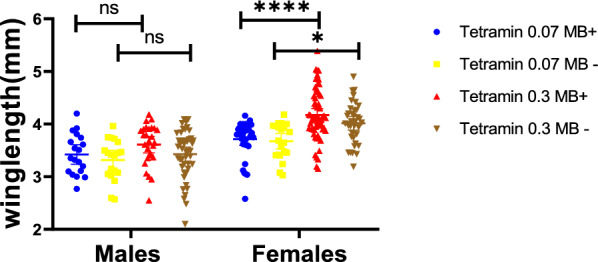
Effects of *Microsporidia MB* on wing length of IMLs under different larval diet quantity regimes. *Microsporidia MB* significantly increased the length of female wings both under the Tetramin 0.07 and 0.3 mg/larva/day diet, respectively. The line and the bars in the middle of each treatment indicate the mean with 95% CI, respectively.

Furthermore, the sizes of the female *Microsporidia MB* IML mosquitoes were significantly larger compared to the control IMLs (uninfected IMLs from the same infected mother) when fed the TD 0.3 mg/larva/day diet (*X*^2^= 23, *df* = 1, *P*< 0.0001; Fig. 6); male mosquitoes on the same diet did not show any difference in size (*U*_(64)_ =  3.58, *P* = 0.115).

**Fig. 7:**
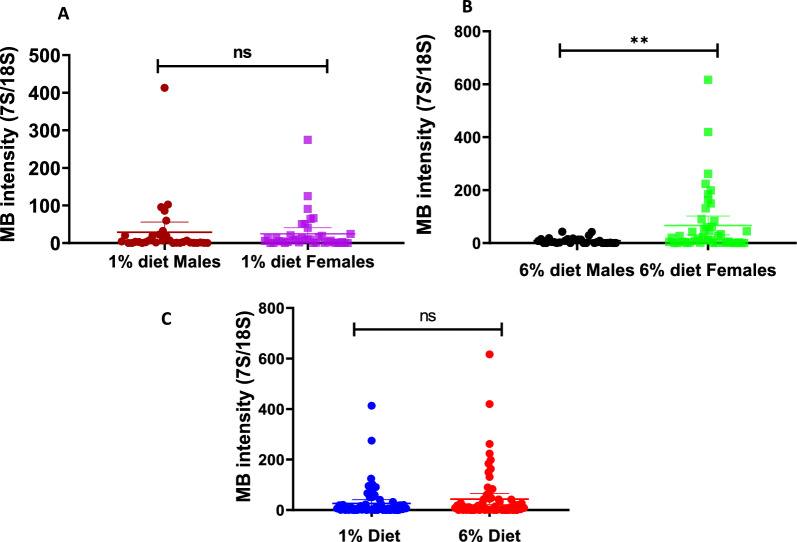
*Microsporidia MB* intensity under different adult diet quantities. **a** Effect of low adult diet (1% glucose) on *Microsporidia MB* intensity in male and female IMLs mosquitoes. **b** Effect of high adult diet (6% glucose) on *Microsporidia MB* intensity in male and female IMLs mosquitoes. **c** Effect of low (1% glucose) and high (6% glucose) diet on the intensity of *Microsporidia MB.* Bars represent the mean with a 95% confidence interval of the *Microsporidia MB* intensities of each diet regime. IMLs, Isofemale lines

There was no significant difference in *Microsporidia MB* intensity between adult mosquitoes fed on the 1% and 6% glucose diet regimes *U*_(76)_= 8.08, *P* = 0.445; Fig. 7c). Under the low nutritional diet (1%) glucose, there was no significant difference in *Microsporidia MB* intensity between male and female mosquitoes (*U*_(39)_= 7.4, *P* = 0.430). In contrast, in adult mosquitoes fed the the 6% diet, *Microsporidia MB* intensity was significantly higher in the female IMLs than in the male IMLs (Fig. 7b; *U*_(46)_= 12.57,* P* < 0.009).

## Discussion

Despite being harboured inside the cells of their hosts, insect endosymbionts are affected by the environmental conditions experienced by their hosts. In this study we investigated how host nutrient availability affects the interaction between *An. arabiensis* mosquitoes and *Microsporidia MB*. Understanding the dynamics of this interaction is important for predicting the ability of *Microsporidia MB* to spread in natural *An. arabiensis* populations and for establishing optimal methods to rear *Microsporidia MB-*infected *An. arabiensis* mosquitoes [21, 46].

Some life history traits were not affected by either *Microsporidia MB* or diet. The prevalence of *Microsporidia MB* was not affected by diet although diet conditions could play an essential role in ensuring successful transmission with a significant increase in symbiont intensity. The results of the intensity experiments showed that in the absence of the TD 0.3 mg/larva/day diet regime, larvae raised on the GD 0.3 mg/larva/day diet can be used to achieve a comparable high *Microsporidia MB* intensity in the mosquito host. The low-intensity results seen under the TD 0.07 mg/larva/day and CD diets could be attributed to the low protein content by quantity and composition, respectively.

Firstly, *Microsporidia MB* reduced larval mortality across the four larval diet regimes, although the impact was more prominent under the TD 0.3 and GD 0.3 mg/larva/d diets. A similar trend was seen for larval development where larvae unde the TD 0.3 g/larva/day diet regime showed the fastest larval development, followed (in order of increased developmental time) by those on the GD 0.3 mg/larva/day diet, the CD diet and finally the TD 0.07 mg/larva/day diet. *Microsporidia MB*-colonised *An. arabiensis* larvae pupated 1.75 days faster than the control larvae without the symbiont under the TD 0.3 mg/larva/day regime; however under the TD 0.07 mg/larva/day regime the development time was delayed and there was no significant difference between *Microsporidia MB*-positive and -negative groups, indicating the impact of diet quality and quantity on development [47, 48]. Larval diet conditions impacted the adult emergence of mosquitoes, although *Microsporidia MB* did not affect the emergence of adults from pupae under the four diet regimes. These findings are similar to those reported in Herren et al. [37]*.*

Under the different larval diets, larvae fed on the TD 0.3 mg/larva/day diet had the lowest larval mortality, fastest larval development and highest adult emergence. These results confirm those of earlier research showing that the diet of a mosquito influences the life history characteristics of that mosquito [21]. In terms of diet composition, only the protein content differed significantly among the various diets, with TD having the highest protein content, followed by the BD and CD diets. Hence, the difference seen in mosquito development must be due to the protein content in the TD diet, as supported by earlier observations that medium levels of protein are essential for a bigger size and weight [21]. The results clearly showed that not only the mosquito (host) is affected by diet but the symbiont as well. The *Microsporidia MB*-conferred fitness advantage on mosquitoes is therefore diet-dependent. These results indicate that the TD 0.3 mg/larva/day diet regime is the most suitable nutritional regime for mass rearing of *Microsporidia MB* mosquitoes among the options tested.

The survival of *Microsporidia MB* adult mosquitoes was affected by the quantity of the adult mosquito’s diet quantity. The longer survival of the *Microsporidia MB* adult *An*. *arabiensis* iso-groups compared to the negative groups reaffirms that the *Microsporidia MB* fitness advantage is diet-dependent. This finding is similar an earlier report of the higher survival of *Rickettsia-*infected whiteflies compared to uninfected ones [49]. It is also worth noting that the quantity of sugar in the adult mosquito’s diet did not affect the intensity of *Microsporidia MB* in the mosquitoes; rather, it affected the presence of *Microsporidia MB*, with the diet condition determining the fitness benefit to the host.

In the IMLs, larval diet quantity significantly affected the intensity of *Microsporidia MB* in adult female mosquitoes but not in adult males. One explanation could be the difference in protein profiles between male and female mosquitoes, with 682 and 422 exclusively different proteins in females and males, respectively [50]. Vertically transmitted symbionts strategically manipulate the female host to ensure their effective transmission to the next generation by various mechanisms [51, 52]. Hence, the higher intensity of *Microsporidia MB* in the females both under the TD 0.3 mg/larva/day diet and compared to males may be ensuring that the symbiont is successfully transmitted to the offspring. The size of mosquitoes was affected by both diet quantity and sex of the mosquito. Research has proven that female mosquitoes with larger body sizes are more likely to become gravid than those with smaller body size [53]. Furthermore, Larger females lay more eggs and undergo shorter gonotrophic cycles than small ones [43, 54, 55]. The quantity of the larval diet affects the size of the mosquito [43], as also noted in the present study. The corresponding higher *Microsporidia MB* intensity with larger body size of *Microsporidia MB*-positive females in the IMLs confirms the reproductive advantage conferred to the host by the symbiont. A maternally transmitted *Rickettsia* symbiont in whitefly causes similar biases in the female as a reproductive surety [49].

Interestingly, adult diet quantity did not affect *Microsporidia MB* intensity. The significantly higher female *Microsporidia MB* intensity compared to that in the male reflects the synergistic impact of both a high larval diet (TD 0.3 mg/larva/day) and a high adult diet (6% glucose).

Insect hosts regulate the intensity of obligate intracellular nutritional symbionts depending on the nutritional status of the host [56]. Under limited thiamine conditions, obligate mutualist *Wigglesworthia* intensity in *Glossina morsitans* increased while an enriched thiamine diet significantly decreased the intensity of the symbiont [34]. As a nutritional symbiont, *Wigglesworthia* synthesises thiamine, hence under an enriched diet, the intensity of the symbiont is reduced by the host since there is no need for nutrient provision [57]. Another experimental manipulation of nutrition in *Acyrthosiphon pisum* (pea aphid) showed an increased obligate *Buchnera aphidicola* population intensity with a corresponding increase in the size of the host when fed on a rich nitrogen diet [58]. The proportional increase in *Buchnera* was to increase nutrient provision by the symbiont to compensate for the growth of the insect. *Wolbachia pipientis*, an intracellular proteobacterium that infects about 60% of insects, is both maternally and horizontally transmitted [56]. It has been demonstrated that the ability of *Wolbachia* symbiont to induce recombination in *Drosophila melanogaster* is titer-dependent [56].

The results from this study demonstrate that *Microsporidia MB* is not a nutritional symbiont. Consequently, there was a resultant lack of directly proportional changes in intensity according to diet quantity [33]. Insect host regulation of symbionts could be responsible for the restriction of the *Microsporidia MB* fitness benefit to *An. arabiensis* under very limited diet conditions, as all resources are channelled to host-symbiont survival [33]. However, under normal diet conditions, the metabolism of the symbiont is not restricted, leading to a full expression of fitness advantage seen in both larval and adult diets. Our results also showed clearly that there was no significant fitness cost to the host even under very limited diet conditions.

## Conclusions

*Microsporidia MB* has no negative effect on the development of *An. arabiensis* even under low diet conditions, which are often experienced in nature, and hence can be used as an effective malaria control tool. The Tetramin 0.3 mg/larva/day diet regime results in a high *Microsporidia MB* intensity in the mosquito. The longer survival of the adult *Microsporidia MB* mosquito fed on 6% glucose is an added advantage for the proliferation of *Microsporidia MB* in the uninfected mosquito population. The diet regimes can, therefore, be utilised for mass rearing of *Microsporidia MB*-infected *An. arabiensis*.

### Supplementary Information

**Additional file 1.**Experimental design to determine the effect of different larval diet regimes on *Microsporidia MB* prevalence and intensity and influence on *Microsporidia MB* on >*An. arabiensis* development fitness.

**Additional file 2.** Main nutritional components of the diets.

**Additional file 3.** Experimental design to determine the effect of *Microsporidia* MB on adult mosquito survival under different adult diet regimes.

**Additional file 4.** (A) Experimental design to determine the effect of different larval diet quantity on *Microsporidia* MB intensity in the isofemale line of *An*. *arabiensis* after vertical transmission. (B) Experimental design to determine the effect of adult diet quantity on *Microsporidia* MB intensity in isofemale line of *An*. *arabiensis* after vertical transmission.

**Additional file 5.** Data generated from the study.

## Data Availability

All data supporting the findings of this study are available as Additional file Data 5.

## Abbreviations

*MB*: *Microsporidia MB*
GLs: Group lines
IMLs: Isofemale lines
