# Supplementary figures and images for: Effect of Microsporidia MB infection on the development and fitness of Anopheles arabiensis under different diet regimes
Source: Parasit Vectors. 2024 Jul 9;17:294. doi: 10.1186/s13071-024-06365-8 (PMC11234536; doi:10.1186/s13071-024-06365-8)

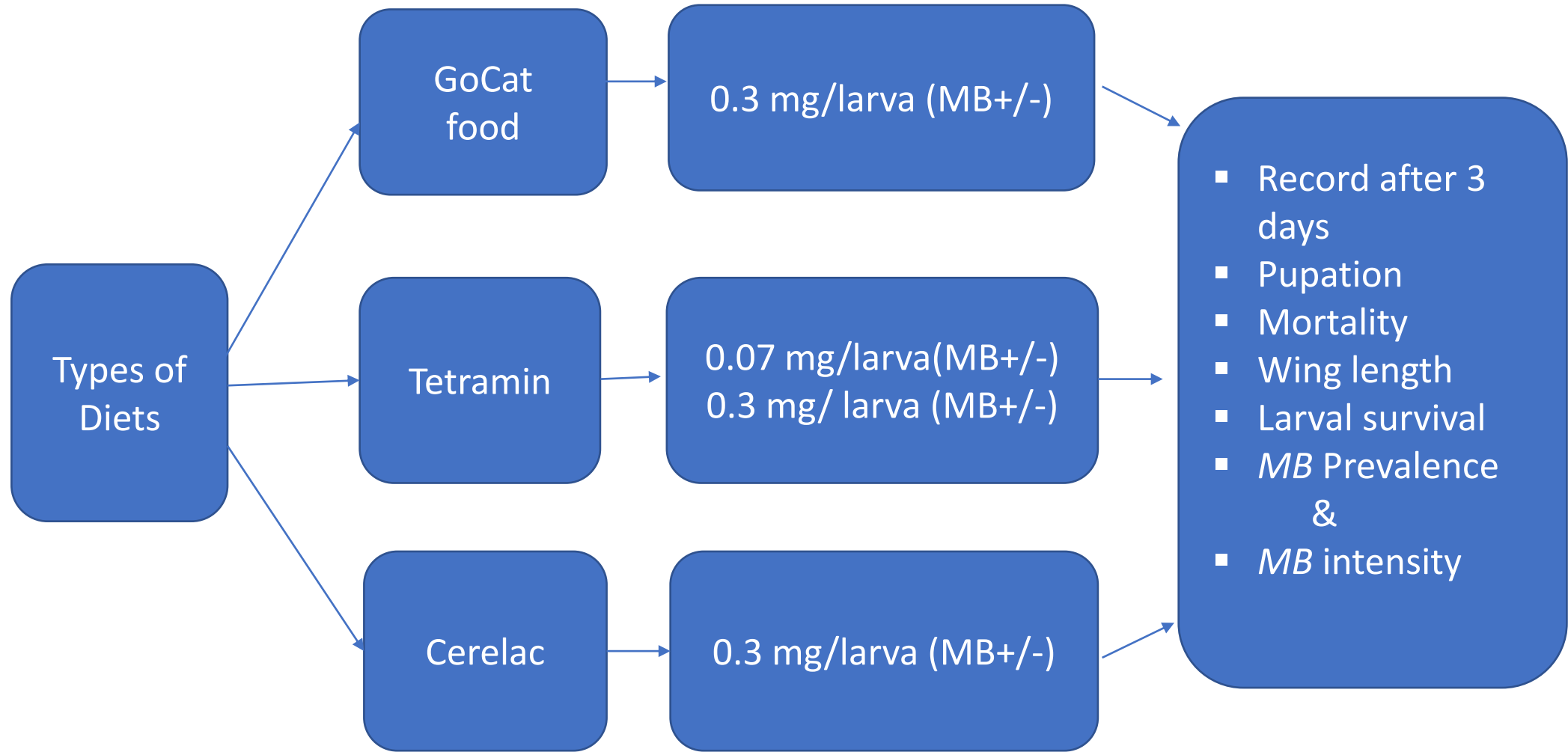

Supplement: Supplementary file 1 — Additional file 1.Experimental design to determine the effect of different larval diet regimes on Microsporidia MB prevalence and intensity and influence on Microsporidia MB on >An. arabiensis development fitness. [file 13071_2024_6365_MOESM1_ESM.pdf]

**A**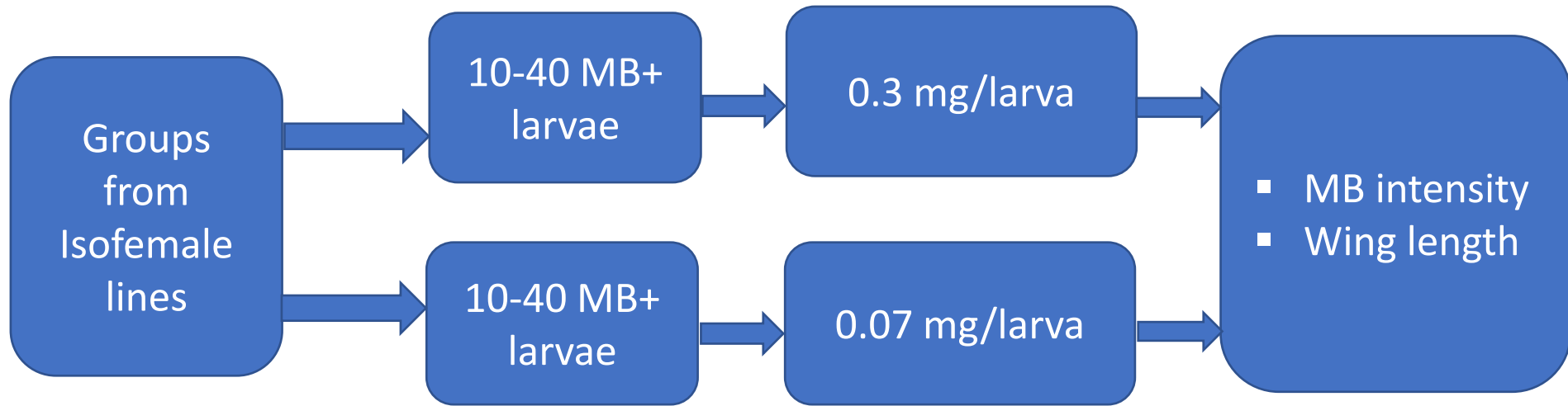**Larval diet****Adult diet****Record****B**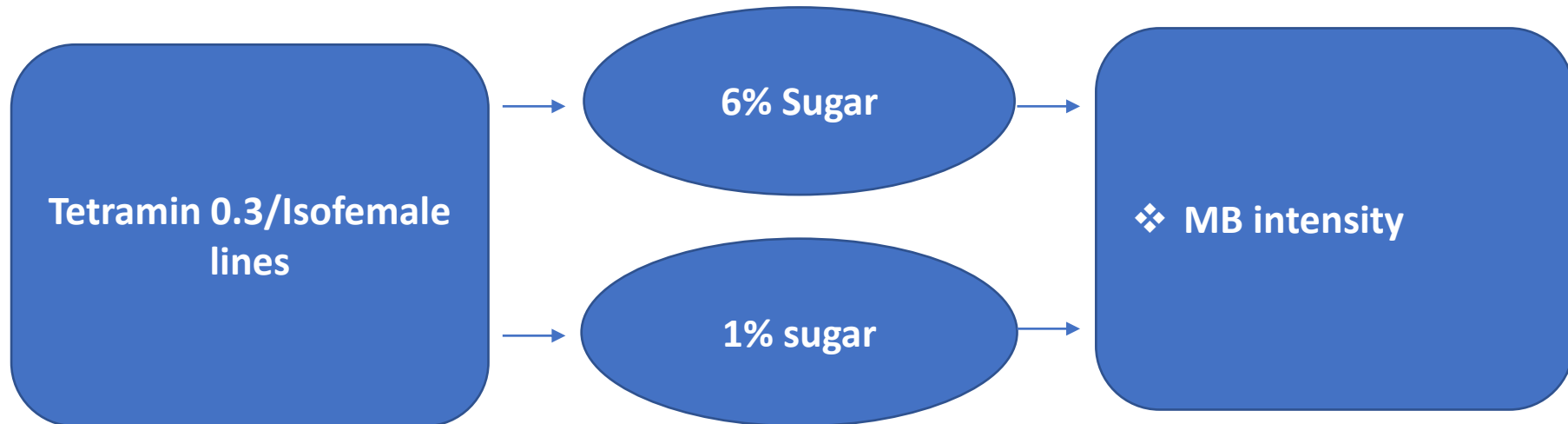

Supplement: Supplementary file 4 — Additional file 4. (A) Experimental design to determine the effect of different larval diet quantity on Microsporidia MB intensity in the isofemale line of An. arabiensis after vertical transmission. (B) Experimental design to determine the effect of adult diet quantity on Microsporidia MB intensity in isofemale line of An. arabiensis after vertical transmission. [file 13071_2024_6365_MOESM4_ESM.pdf]
