# Supplementary material for: Effect of Microsporidia MB infection on the development and fitness of Anopheles arabiensis under different diet regimes
Source: Parasit Vectors. 2024 Jul 9;17:294. doi: 10.1186/s13071-024-06365-8 (PMC11234536; doi:10.1186/s13071-024-06365-8)
Supplement: Supplementary file 2 — Additional file 2. Main nutritional components of the diets. [file 13071_2024_6365_MOESM2_ESM.pdf]

Main Nutritional Composition of diet

Cerelac		Gocat		TetraMin	
Protein	14%	Protein	30%	Protein	46%
Fat content	9.6%	Fat content	10%	Fat content	11%
Fiber total	1.5%	Crude fibre	3%	Crude fiber	2%
