# Supplementary material for: Effect of Microsporidia MB infection on the development and fitness of Anopheles arabiensis under different diet regimes
Source: Parasit Vectors. 2024 Jul 9;17:294. doi: 10.1186/s13071-024-06365-8 (PMC11234536; doi:10.1186/s13071-024-06365-8)
Supplement: Supplementary file 3 — Additional file 3. Experimental design to determine the effect of Microsporidia MB on adult mosquito survival under different adult diet regimes. [file 13071_2024_6365_MOESM3_ESM.pdf]

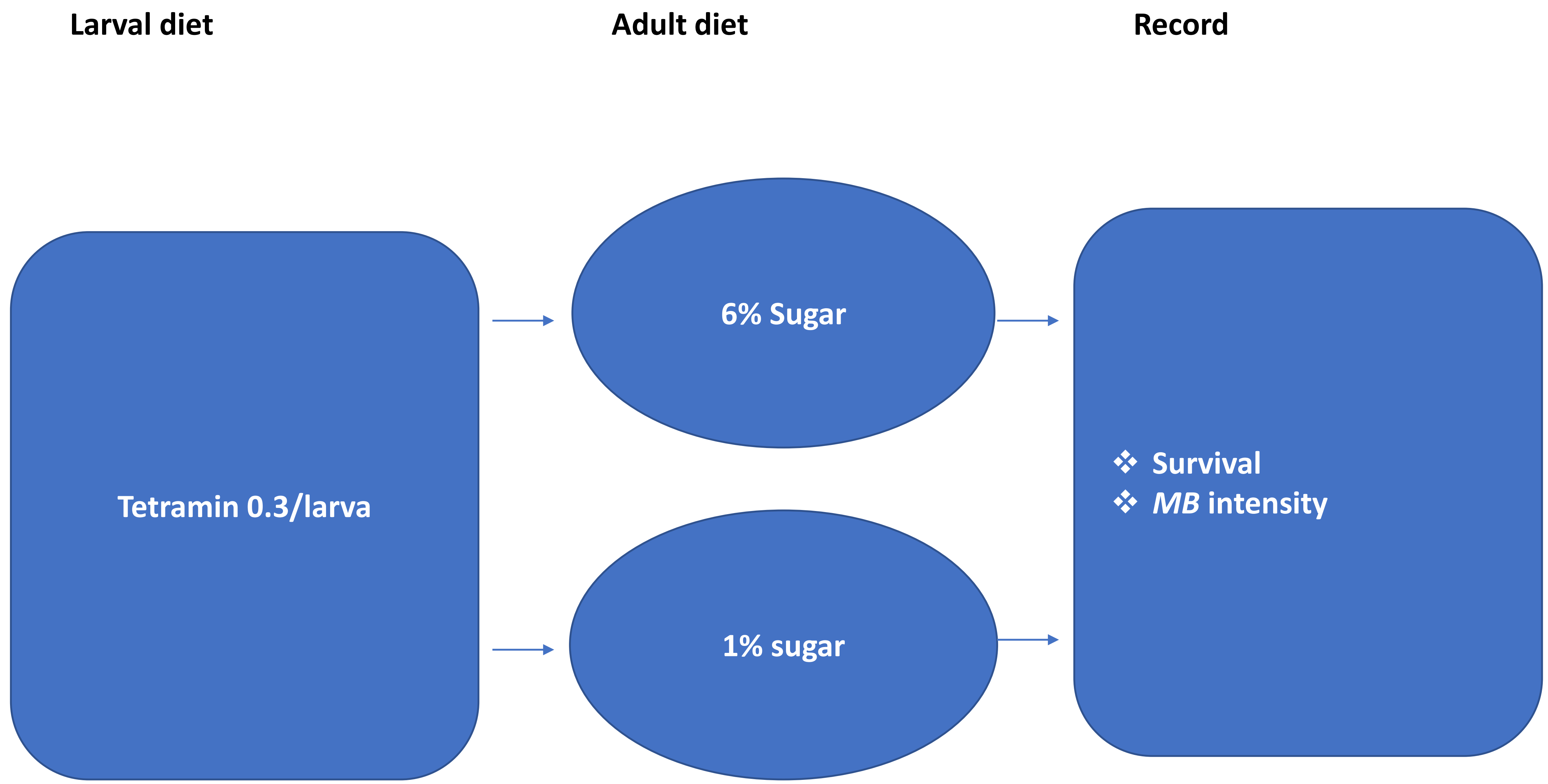
Larval diet
Adult diet
Record
Tetramin 0.3/larva
6% Sugar
1% sugar
❖ Survival
❖ *MB* intensity
